# Identifying new variation at the *J* locus, previously identified as *e6*, in long juvenile ‘Paranagoiana’ soybean

**DOI:** 10.1007/s00122-020-03746-2

**Published:** 2021-01-02

**Authors:** Nour Nissan, Elroy R. Cober, Michael Sadowski, Martin Charette, Ashkan Golshani, Bahram Samanfar

**Affiliations:** 1 Ottawa Research and Development Center, Agriculture and Agri-Food Canada, Ottawa, ON Canada; 2 Department of Biology, Ottawa Institute of Systems Biology, Carleton University, Ottawa, ON Canada

## Abstract

**Key message:**

**A previously identified soybean maturity locus, *E6***,** is discovered to be ***J*,** with the long juvenile allele in Paranagoiana now deemed *j***−***x***.

**Abstract:**

Soybean grown at latitudes of ~20° or lower can produce lower grain yields due to the short days. This limitation can be overcome by using the long juvenile trait (LJ) which delays flowering under short day conditions. Two LJ loci have been mapped to the same location on Gm04, *J* and *E6*. The objective of this research was to investigate the *e6* allele in ‘Paranagoiana’ and determine if *E6* and *J* are the same locus or linked loci. KASP markers showed that *e6* lines did not have the *j−1* allele of LJ PI 159925. A population fixed for *E1* but segregating for *E6*, with *e6* introgressed from Paranagoiana, showed single gene control for flowering and maturity under short days. Sequencing Glyma.04G050200, the *J *gene, with long amplification Taq found that the *e6* line ‘Paranagoiana’ contains a *Ty1-copia* retrotransposon of ~10,000 bp, inserted within exon 4. PCR amplification of the cDNA of Glyma.04G050200 also showed differences between the mRNA sequences (presence of insertion in *j*−*x*). Hence, we conclude that the loci *E6* and *J* are one locus and deem this new variation found in Paranagoiana as *j−x*.

**Supplementary Information:**

The online version contains supplementary material available at 10.1007/s00122-020-03746-2.

## Introduction

Soybean [*Glycine max* (L.) Merr.] is an economically valuable legume crop grown world-wide for human consumption (protein and vegetable oil), animal feed and industrial product uses. It is also an important player in sustainable agricultural practices due to its nitrogen fixation capabilities (Boerema et al. 2016; Sedivy et al. 2017).

The soybean genome is complex due to two whole genome duplication events that result in nearly 75% of soybean genes being present in multiple copies (Du et al. 2010). The ~ 1.1 GB genome is also made up of highly repetitive sequences such as transposable elements (TEs) adding to the complexity (Gao et al. 2014; Schmutz et al. 2010; Xie et al. 2019).

Transposable elements are mobile and have the ability to “jump” and insert themselves into different areas of the genome. They make up a large percentage of flowering plants genomes and are comprised of two types: DNA transposons and retrotransposons (RTs) (Gao et al. 2014). DNA transposons could be subdivided into a minimum of 10 subfamilies while retrotransposons are made up of long terminal repeat (LTR) and non-LTR RTs (Tian et al. 2012). Due to their large presence in eukaryotic genomes, retrotransposons can take part in regulating the expression of genes, altering their function as well as creating new genes (Du et al. 2010).

Soybean is a short-day plant that is cultivated over latitudes ranging from 35° S to 50° N. Short days (12 h of day-length or less) allow the plant to flower and mature quickly. Phenology is delayed in long day conditions resulting in lower yields if maturity is not reached before frost, while in short days flowering can occurs early resulting in short low yielding plants (Kong et al. 2014; Miranda et al. 2020). Photoperiod sensitivity in soybean plays a key role in determining adaptation for a specific latitude by controlling time to flowering and maturity. Hence, local adaptation is essential for successful seed production and seed quality (Li et al. 2017; Tsubokura et al. 2013). Soybean’s ability to adjust to a range of latitudes and conditions is due to natural variations in maturity genes and quantitative trait loci (QTL’s) controlling time to flowering and maturity (Kong et al. 2014).

Twelve maturity loci in soybean have been discovered and assigned as *E* loci. *E1* (Glyma.06g207800) and *E2* (Glyma.10g221500) (Bernard 1970); *E3* (Glyma.19g224200) (Buzzell 1971); *E4* (Glyma.20g090000) (Buzzell and Voldeng 1980), *E6* (Bonato and Vello 1999), *E7* (Cober and Voldeng 2001); *E8* (Cober et al. 2010); *E9* (Glyma.16g150700) (Kong et al. 2014); *E10* (Glyma.08g363100) (Samanfar et al. 2017), *E11*(Wang et al. 2019), *Dt1* (Glyma.19g194300) (Liu et al. 2010; Tian et al. 2010) and the *JUVENILE* gene *J* (Glyma.04G050200) (Ray et al. 1995). *E1* to *E4*, *E7*, *E8* and *E10* alleles delay flowering under long day conditions, while *E9* and *E11* alleles promote flowering under long day conditions. Underlying genes for *E1* to *E4*, *E9*, *E10* and *J* have been determined through different mapping as well as molecular biology related approaches (Liu et al. 2008; Samanfar et al. 2017; Watanabe et al. 2011; Xia et al. 2012; Zhao et al. 2016).

Soybean cultivation below 20° was hindered by extremely low grain yields before the discovery of the long juvenile (LJ) trait (Miranda et al. 2020). The LJ trait, which became important in South America in the 1970s, expanded soybean production to tropical latitudes below 20˚ and even as far as the equator (Lu et al. 2017). This trait was first identified in PI 159925 from Peru controlled by a single locus *J* (Glyma.04G050200) (Hartwig and Kiihl 1979; Ray et al. 1995), and also in Brazil with cultivar Paranagoiana, controlled by the locus *E6*, a natural variant of the cultivar Parana (Bonato and Vello 1999). In PI 159925, the source of the LJ trait is a (C/−) indel within exon 4 of *J*, causing a frameshift and leading to the *j−1* mutation (Hartwig and Kiihl 1979; Ray et al. 1995). However, in Paranagoiana, the (C/−) indel does not exist (Li et al. 2017). The introduction of the LJ trait made it possible to improve yield by extending the vegetative phase to ensure sufficient growth under short-day conditions (Lu et al. 2017; Yue et al. 2017).

Previous research discovered nine different alleles for *J* (*J*, *j−1* to *j−8*) all except for *J* of which are loss-of-function mutations found across the global soybean germplasm. These recessive alleles displayed delayed flowering and increased plant height, node number, and grain yield under inductive short-day conditions (Lu et al. 2017).

*J* is an ortholog of the *Arabidopsis thaliana EARLY FLOWERING 3 (ELF3)* gene (Yue et al. 2017). *J* expression is under the influence of *E1*, which is a legume-specific flowering repressor. The interaction between the two genes relieves repression of two important *FLOWERING LOCUS T (FT)* genes and promotes flowering under short days. This occurs due to *J* physically associating with the promotor of *E1*, and downregulating its transcription (Lu et al. 2017).

*E6* was mapped to the same region as *J* on chromosome 04 between SSR markers Sat_337 and Satt396 (Li et al. 2017). Up until this point, *E6* and *J* have both been hypothesized to be involved in the LJ trait due to a 1:15 segregation ratio seen in segregating populations (Carpentieri-Pipolo et al. 2002; Cober 2011). However, the underlying gene responsible for *E6* is yet to be identified.

The objective of this study was to attempt to determine the underlying gene for *E6* and to address whether *J* and *E6* are different loci.

## Materials and methods

### Plant materials, DNA extraction, PCR and primers

The *e6* allele was introgressed from Paranagoiana into an early maturity Harosoy isoline, OT94-47. The development of the backcross three line (X5063-39) was described by Cober (2011). Back crossing (BC) was continued to develop the BC_5_ line X5683-33 with selection for late flowering under 12 h photoperiods. Population X6055 was developed from the cross X5683-33/OT94-51, which is a population fixed for *E1* but segregating for *E6*. OT94-51 is also an *E1e3e4* Harosoy isoline. The X6055 F_2_ population was phenotyped for phenology under 12 h photoperiod.

Frozen young trifoliate leaves harvested from soybean grown in a greenhouse were used for DNA extraction with the urea extraction technique described by Molnar et al. (2003). The gene was divided in two parts, and both parts were amplified independently in order to allow proper amplification to take place. PCR conditions used to amplify the first half of *J* were one cycle of 2 min at 94 °C; 30 cycles of 30 s at 94 °C, 20 s at 58 °C and 4 min 30 s at 72 °C, using High Fidelity Platinum® Taq DNA Polymerase. In order to amplify the insertion within exon 4 of *J*, TaKaRa LA (Long Amplification) Taq® DNA Polymerase (Mg^2+^plus buffer) was used at conditions of one cycle of 1 min at 94 °C; 30 cycles of 10 s at 98  C, and 15 min at 60°°C, with a final extension of 10 min at 72 °C. The PCR reactions were confirmed by gel electrophoresis, and imaged using SYBR safe and blue light on a 1% agarose gel, with a GeneRuler 1 kb Plus DNA Ladder. Primer3 online software (www.bioinfo.ut.ee/primer3) was used for primer design. Entire sets of primers used to amplify the gene *J* were purchased from Integrated DNA Technology (IDT), Coralville, Iowa, United States (IDT. Inc). Primers are listed in Supplementary File 1, Table 1.

### Sequencing *J* and *j−x*

Two sequencing methods were used to sequence *J* and the *j−x* insertion. Sanger sequencing was performed in conjunction with BigDye Terminator v3.1 Cycle Sequencing Kit (Life technologies, Carlsbad, California, USA) as per manufacturer’s instructions. Samples were run on an ABI 3130xl automated sequencer (Applied Biosystems) in-house at AAFC-ORDC, Ottawa. Primers used are included in Supplementary File 1, Table 1.

To sequence the unknown ~10 Kbp insertion in *j−x*, a primer walking approach was used. First, specific primers were used (2F and 2R) from the known regions of the gene on either side of the insertion to sequence ~800 base pairs at a time until the entire insertion was sequenced. The complete sequence is provided in Supplementary File 2 (GenBank ID: MT633125) Once the first ~800 bases were identified, new primers were generated complementary to the last ~20–30 bases and used to sequence the next ~800 unknown bases on either end. This was performed until the entire insertion was sequenced. Primer walking was performed in Genome Quebec research facilities. A list of primers is presented in Supplementary File 1.

#### KASP

The single nucleotide deletion (indel) (C/−) showing the *j−1* mutation was used in designing the Kompetitive Allele-Specific PCR (KASP) assay per the KASP manual (LGC Genomics, 2013). One common reverse primer, as well as two allele-specific forward primers, was designed by LGC genomics (Supplementary File 1, Table 2). The PCR products were viewed using a Tecan Spark microplate reader, and the data was analyzed using KlusterCaller software (LGC Genomics).

### Total RNA extraction and purification

The same young frozen trifoliate leaf samples which were used for DNA extraction were utilized for RNA extraction. RNA was extracted from lines Harosoy, Parana, and Paranagoiana using a Trizol method (Thermo Fisher Scientific Inc., Wilmington, DE, USA) as per manufacturers instruction, with the addition of phase lock tubes (Lexogen, Greenland, NH, USA) for better separation. Finally, a digestion step using DNase I (Thermo Fisher Scientific Inc., Wilmington, DE, USA) to remove contaminating DNA was performed as a final step. RNA integrity was checked by using Nanodrop 2000 spectrophotometer (Thermo Fisher Scientific Inc., Wilmington, DE, USA) as well as 1% agarose gel electrophoresis, and imaged using blue light.

### cDNA synthesis and PCR amplification

Purified RNA was used to construct a full cDNA library using the iScript Select cDNA Synthesis Kit as per manufacture instructions using 1 μg of RNA (Bio-Rad Laboratories, Hercules, CA, USA). The cDNA library was used for PCR amplification of *J* using the same PCR conditions as the genomic DNA. Proper cDNA synthesis was ensured when samples were amplified and ran on 1% agarose gel electrophoresis to produce a single specific product.

### Allele-specific markers

The marker for determining the *j−x* allele is a PCR-based allele-specific marker using 2F and 2R primers (Supplementary File 1, Table 1). The band produced for *J* is shown at ~1500 bp and the one at ~10,000 is the *j−x* allele. Please note that in order to have a proper amplification and to ensure strong band signals, TaKaRa LA Taq® DNA Polymerase (Mg^2+^plus buffer) must be used.

## Results

### KASP for *j−1* allele

**Fig. 1:**
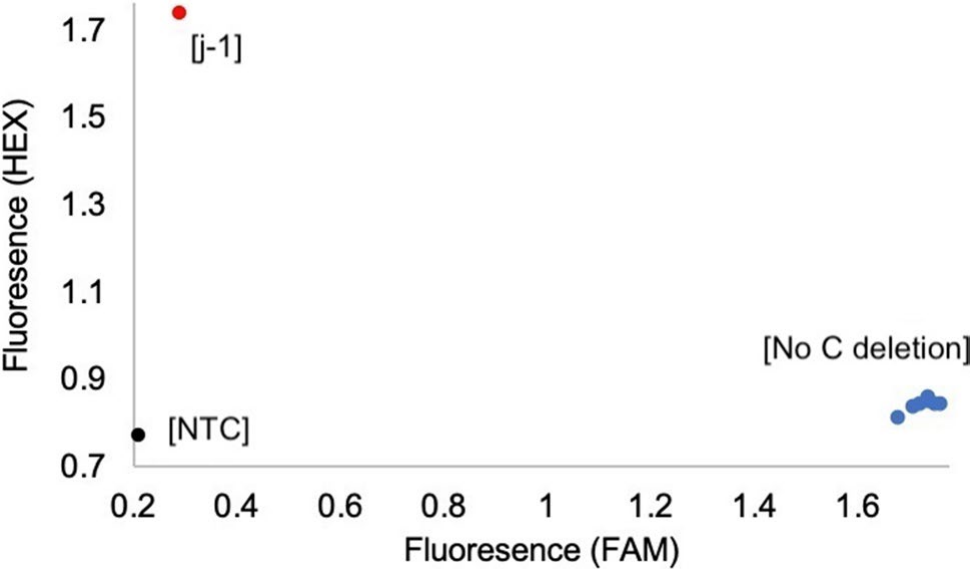
Results of KASP assay screening for *j−1* allele. *j−1* (indel) was screened using FAM and HEX fluorescent fluorophores. Shown in red is the soybean line representing the C nucleotide deletion presented by HEX fluorophore (PI 159925), while the rest of the control and *e6* lines are shown in blue representing no C nucleotide deletion, presented by FAM fluorophore fluorescence (AC Colibri, Harosoy, OT94-47, Williams 82, Parana, Paranagoiana and X5063-39). Finally, the no template control (NTC) is shown in black correctly displays no florescence (color figure online)

KASP assay was performed to assay the *e6* lines for the *j−1* allele found in PI 159925 (Lu et al. 2017). KASP results indicated that the *j−1* allele was not present in the control lines nor the *e6* lines, and that it was only present in PI 159925 (Fig. 1).

### Sequencing *J*

**Fig. 2:**
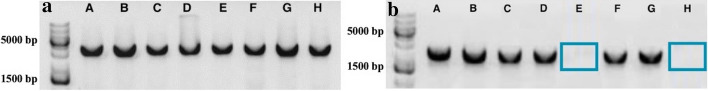
Amplifying Glyma.04g050200. **a** PCR product for the first half of *J* up until exon 3 ~3700 bp in length. **b** PCR product for the second half of *J* exon 3 until end of exon 4 ~2300 bp in length. Lines used: (**a**) AC Colibri, (**b**) Harosoy, (**c**) OT 94–47, (**d**) Parana, (**e**) Paranagoiana, (**f**) PI 159925, (**g**) Williams 82, (**h**) X5063-39. The blue rectangles highlight the two *e6* lines which failed to amplify within the second half of the gene. Samples ran on a 1% agarose gel with a GeneRuler 1 kb Plus DNA ladder, and were imaged using SYBR safe and blue light (color figure online)

A number of lines were genotyped at the *J* locus by sequencing the underlying gene Glyma.04g050200. AC Colibri, Harosoy, OT94-47, Williams 82, and Parana showed a consistent pattern across the conventional juvenile lines. PI 159925 displayed the (C/−) indel, the *j−1* allele, while the rest of the sequenced lines exhibited no polymorphisms (data not shown). The gene was split in two to allow for proper amplification. The first and second halves of the amplified regions are shown in (Fig. 2a, b). An obstacle was encountered when amplifying the second half of Glyma.04g050200 gene in the two *e6* lines: Paranagoiana and X5063-39 (OT94-47 backcrossed three times to Paranagoiana with selection for late flowering under 12 h photoperiod). Up until exon 3, *J* was amplified without any issues and with no variation from the conventional juvenile lines (Fig. 2a), however, further amplifications failed for the rest of the gene in the two *e6* lines (Fig. 2b). Due to this issue, multiple PCR conditions and primer combinations were examined in attempts to amplify the gene in those two lines. Nonetheless no procedure was successful.

**Fig. 3:**
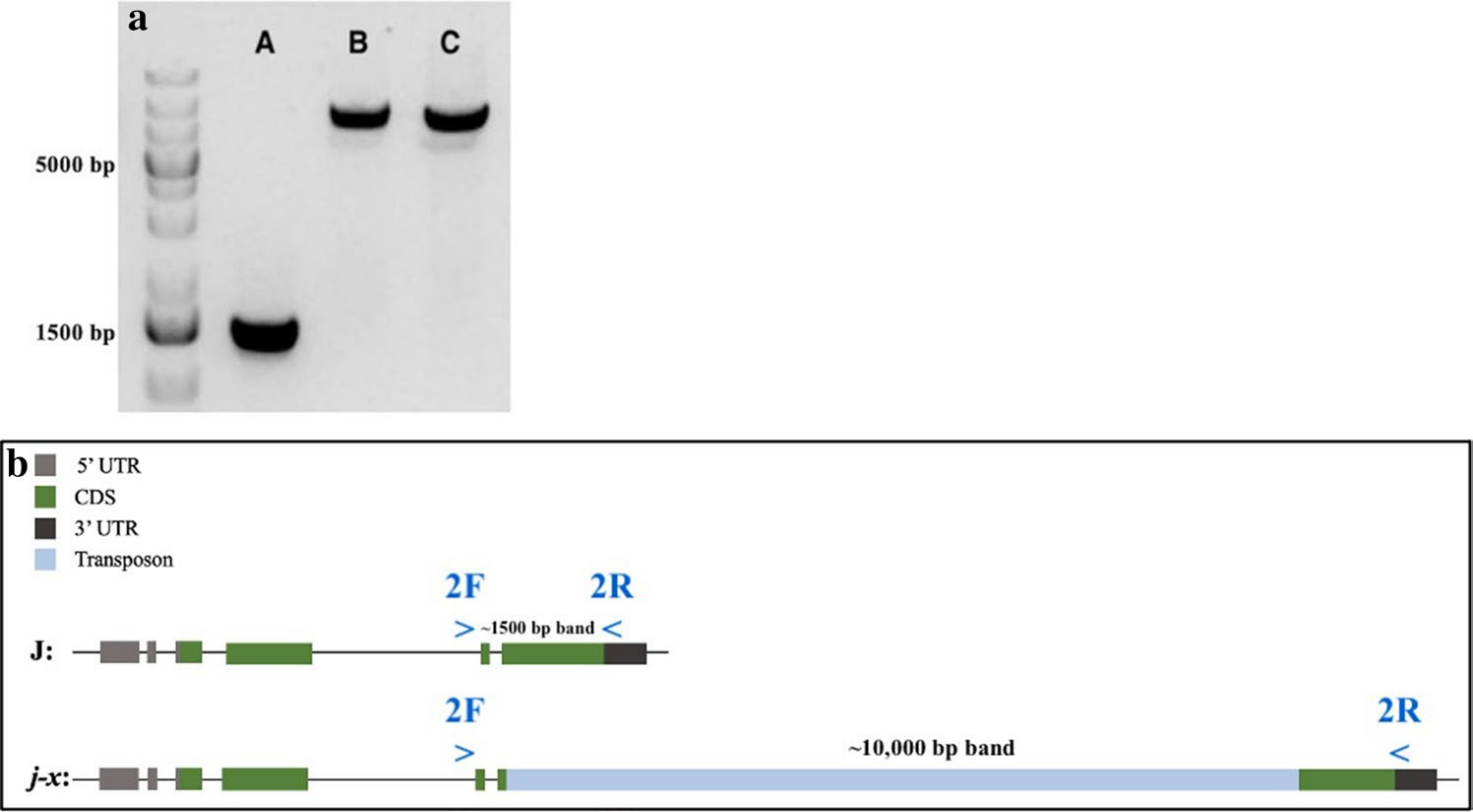
Amplification of insertion in *j−x* and its location**. a** PCR product for the second half of *J* containing exons 3 and 4 in lines (**a**) OT 94–47 (control) ~1,500 bp in length, (**b**) Paranagoiana and (**c**) X5063-39 (*j−x*, previously presumed *e6* lines) ~10,000 bp in length, using TaKaRa LA Taq. Samples ran on a 1% agarose gel and imaged using SYBR safe and a blue light with a GeneRuler 1 kb Plus DNA Ladder. **b** Glyma.04g050200 gene *J* and *j−x* compared and showing the location of the insertion within exon 4 of *j−x.* In blue, primer combination used is shown as well as their location within the gene (color figure online)

PCR reactions were then carried out using TaKaRa LA Taq®, based on previous PCR amplification procedures (Kong et al. 2010). An amplified region for both *e6* lines with a band at ~10,000 bp, and a control band at ~1500 bp were found (Fig. 3a). In order to confirm the results and to rule out potential false positive results due to non-specific binding, the ends of the new amplicons were sequenced and found to match the sequence of *J,* implying that the *e6* lines may carry a large insertion in either exons 3 or 4 of *J*.

### Primer walking to sequence and identify insertion

To determine the sequence of this large insertion in *J*, a primer walking approach was undertaken at the Genome Quebec research center. Results from primer walking indicated that both *e6* lines possessed a novel allele at the *J* locus which was different from the other eight non-functional alleles (*j−1*,…, *j−8*) (Lu et al. 2017). A large insertion was found in exon 4 of the gene (Fig. 3b).

The ~10,000 bp insertion was investigated in NCBI conserved domains and found to include an integrase core domain, a GAG-pre-integrase domain, reverse transcriptase, ribonuclease H enzyme (RNase HI), a zinc knuckle, and an LTR-polyprotein or retrotransposon. The integrase core domain allows the integration of a DNA copy of the viral genome into the host chromosome, while the GAG-pre-integrase domain is known to be associated with retroviral insertion elements (Jakowitsch et al. 1999; Wright & Voytas 2002). RNAse H is the endonuclease which cleaves the RNA/DNA hybrid, which in retrotransposons also performs degradation of the original RNA template as well as a few other housekeeping roles (Malik 2005). The zinc knuckle is a zinc binding motif mainly composed of gag proteins, and finally, the LTR-polyprotein or retrotransposon is found in plants and fungi (Wright and Voytas 2002). Based on the results from NCBI conserved domains (https://www.ncbi.nlm.nih.gov/Structure/cdd/wrpsb.cgi), the insertion codes for an LTR-retrotransposon of the *Ty1-copia* family. The full sequence of *j-x* was determined, and is available in the Supplementary File of this paper as well as on GenBank (Supplementary File 2, GenBank ID: MT633125).

### RNA extraction and cDNA amplification results

RNA was extracted from 2 control *J* and *E6* lines (Harosoy and Parana) respectively, and one *e6* line (Paranagoiana) in order to confirm the retrotransposon’s presence in the mRNA. Once the extraction was complete, a cDNA library was created for amplification of the *J* gene. Primer combinations (SF2-SR3) and (SF3-SR6) (listed in Supplementary File 1, Table 1.) were used to amplify a section in the first half (identical in contrasting lines for *E6*) and the second half of the *J* gene (different between *E6* and *e6* due to the insertion). Supplementary Figure 1a represents the first portion of *J* amplified, while the second portion of *J* containing the insertion is represented in Supplementary Figure 1b. As seen, the *j−x* line, Paranagoiana, has a band for the first half of the *J* gene in the amplified cDNA, however, the band is absent in the second half containing the insertion (Supplementary Figure 1a, b). New primers were developed spanning a portion of the insertion (FcDNA2/RcDNA2) which when PCR amplified produced the correct band for the *j−x* line at ~500 bp and no band for the two control lines tested (Supplementary Figure 1c). In order to confirm the results and rule out any improper cDNA transformation or non-specific annealing, two primers (Fran1/Rran1) were used for the amplification of a random gene (Glyma.06g314300) within the cDNA library of all three lines (data not presented).

### Allele-specific marker development for *j−x*

Allele-specific diagnostic markers are valuable in accelerating breeding for the LJ trait. An allele-specific marker has been developed for *J* and *j−x* alleles. In this case, a PCR-based method was designed as a positive diagnostic marker for the large insertion identified in *j−x*. In order to confirm the efficacy of the procedure, TaKaRa LA Taq® should be used followed by the PCR protocol presented in the methods section. Results yield a ~10,000 bp band for the *j−x* allele using primers 2F and 2R (Fig. 3a).

### Long juvenile trait under single gene control

**Fig. 4:**
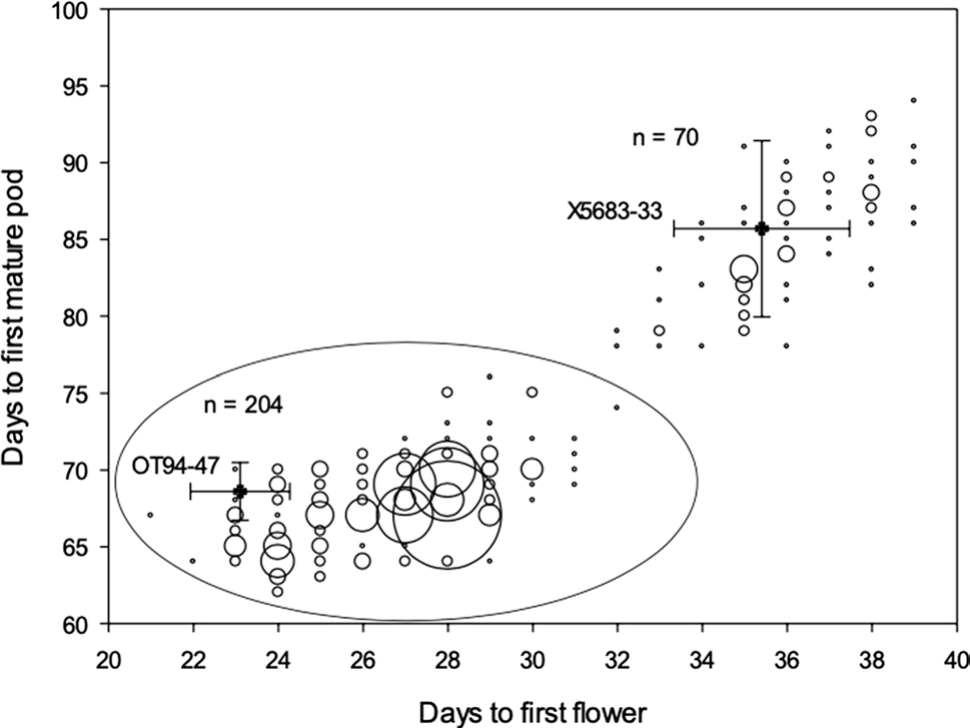
Days to flowering and days to first mature pod for an F_2_ population from a cross between X5683-33 (Harosoy LJ isoline) and OT94-51 (Harosoy *E1e3e4 *isoline). Early lines are enclosed within an ellipse. Bubble size indicates the number of lines with the same phenotype ranging, from 1 to 19. Conventional (OT94-47) and LJ (X5683-33) lines flowering and maturity mean values ±2 standard errors are shown with closed symbols

Since the original work with the introgression of *e6* into an early maturing Harosoy background (Cober 2011) reported that two genes where responsible for the genetic control of the LJ trait, and since the *e6* back cross derived lines were found to have the E1 genotype, cross X6055 was made between the *e6* Harosoy isoline X5683-33 and OT94-51 (Harosoy *E1e3e4*). The population was phenotyped under 12 h days and the population segregated 204 early flowering and maturing and 70 late flowering and maturing (Fig. 4). This fits a 3:1 segregation ratio for a single gene (*n* = 274, *p* = 0.89).

## Discussion

Since *E6* and *J* were mapped to the same region on Gm04 between SSR markers Sat_337 and Satt396 (Bonato and Vello 1999; Li et al. 2017; Ray et al. 1995), we first screened our lines at the *J* locus. *J* (Glyma.04g050200) was identified in PI 159925 with a (C/−) indel, *j*−1 (Hartwig and Kiihl 1979; Ray et al. 1995) while *E6* was known to be a wild type in the cultivar Parana. The LJ cultivar Paranagoiana, *e6*, was selected from Parana (Bonato and Vello 1999; Ray et al. 1995).

In a previous study, nine alleles were found for the gene, Glyma.04g050200. named *J*, *j−*1, *j−*2, *j−*3, …, *j−*8 (Lu et al. 2017). This analysis, however, grouped Paranagoiana and Parana with Williams 82 in the same *J* haplotype when they clearly have different LJ phenotypes (Cober 2011). A recent study described a lack of amplification at the *J* locus in Paranagoiana and labeled it as the *j−x* allele. However, the study did not differentiate between both *J* and *E6* loci (Miranda et al. 2020).

A KASP assay was used to assay for the *j−*1 allele (C/−) indel found in PI 159925. The *j−*1 allele was not found in the *e6* lines (Fig. 1), and our results agreed with the findings from a previous study (Li et al. 2017).

To investigate the possibility of other variations at the *J* locus, Sanger sequencing was used to re-sequence *J* in our lines. During amplification, it was found that the *e6* lines did not produce a band for exons 3 and 4 (Fig. 2b) similar to the non-amplification reported by Miranda et al (2020). By using TaKaRa LA Taq®, an unexpected ~10,000 bp band was produced in the new amplicons for the last 2 exons of the gene in the *e6* lines, while a ~1500 bp band was seen for the wild type lines (Fig. 3a). The inability of the Platinum® Taq to amplify the final 2 exons of *J* in the *e6* lines was determined to be due to the large insertion found in that area. The TaKaRa LA Taq®, however, had the ability to amplify this large region while maintaining specificity which, in turn, unveiled a large insertion within the gene.

*Ty1-copia* retrotransposons are the most abundant class of transposon and have the main domains (GAG), protease (PR), integrase (INT), reverse transcriptase (RT), and ribonuclease H (RNase HI) which were all found in the insertion using NCBI conserved domains (Galindo-González et al. 2016). The sequence is also flanked by LTRs as seen in Supplementary File 2 (GenBank ID: MT633125). Ty1-*copia* elements are able to randomly spread throughout the genome and are associated with altering gene regulation, and even landing within genes and disrupting their functions or altering their splice patterns (Kanazawa et al. 2009; Tsuchiya and Eulgem 2013).

LTR-retrotransposons are highly present in plants, compared to all other eukaryotes, and make up a large proportion of the genome. They are an ongoing source of genetic variation as well as important drivers of interspecies diversity (Huang et al. 2012). Transposable elements have the ability to cause variations in plant phenotypes, such as time to flowering, size of fruit and trichome presence (Bayless et al. 2019). Transposable elements which insert in intronic regions of genes have been known to affect RNA processing. In Arabidopsis, it was found that the insertion of a TE in the first intron of the *FLOWERING LOCUS C (FLC)* gene caused the host to be susceptible to siRNA-mediated transcriptional gene silencing which resulted in reduced expression of the gene and late flowering (Liu et al. 2004). On the other hand, TEs which are inserted into exons of genes have been shown to disrupt the exon, likely disrupting transcription and ending it prematurely due to the TE generating a stop codon (Galindo-González et al. 2018).

By comparing the mRNA for *J* in the control lines and the *j−x* line, we determined that the insertion affected the transcriptome, and that the LJ phenotype is related to the insertion of the retrotransposon in *j−x*. Based on the second amplicon of the cDNA, which attempts to amplify the region encompassing the insertion (end of exon 2 to halfway into exon 4), no band is visible for the *j−x* line while bands were present for the controls (Supplementary Figure 1b). This suggested the possibility of an incomplete mRNA being present in *j−x* with the transcription of the remainder of exon 4 missing. With the use of new primers spanning the first ~500 bp of the insertion, we were able to show that the insertion made it into the mRNA of *j−x,* but that exon 4 is not fully present within the mRNA (Supplementary Figure 1b, c). We verify this by using random primers to amplify a random gene (Glyma.06g314300) within the cDNA library and produce 3 correct bands each at ~600 bp in length for the random gene (data not shown). Hence, through this, we show that the mRNA sequences of *J* and *j−x* differ, in turn, causing a variation in the protein sequence, and likely creating a truncated and dysfunctional protein.

There are occurrences in maturity genes where a retrotransposon leads to a loss-of-function in the protein and hence result in displaying a different phenotype. For example, in *E4* the recessive allele *e4-SORE1* has a retrotransposon in exon 1 of the gene which leads to a pre-mature stop codon and creates a non-functional protein exhibiting photoperiod insensitivity, and leading to early flowering in long day conditions (Liu et al. 2008; Tsubokura et al. 2013). The *e9* recessive allele also has a *SORE-1* insertion within the first intron of the gene which causes it to be a leaky allele, and leads to delayed flowering (Zhao et al. 2016).

While it is clear that the *e6* lines contain a novel allele at the *J* locus, the possibility still remained that *E6* and *J* were different loci. In a previous study, a 15:1 ratio was displayed in an F_2_ population from a cross between OT94-47 and Paranagoiana (Cober 2011). The same ratio was also seen in a cross between OT94-47 and PI 159925 (Cober 2011), and in other crosses (Carpentieri-Pipolo et al. 2002), indicating that two genes may control the LJ trait. In our current population from a cross between conventional and LJ lines evaluated under a 12 h photoperiod condition, lateness was found to be controlled by a single recessive gene (Fig. 4). This population was fixed for the *E1* allele, and only a single gene was segregating for the LJ phenotype. *E1* and *J* interact due to *J* physically associating with the promotor of *E1* (Lu et al. 2017), and both can be seen as controlling the LJ trait.

Since a retrotransposon is present in the genomic DNA of Paranagoiana and X5063-39, and there are differing mRNA amplicons (Supplementary Figure 1), as well as *E6* and *J* being mapped on the same chromosome to the same SSR markers, we can conclude that *E6* and *J* are the same locus, and that the *e6* lines contain a novel allele (*j−x*) of the gene *J*. Previous work (Cober 2011) demonstrated two genes controlled the LJ phenotype, and it was hypothesized that *E6* and *J* may control the LJ phenotype. When it was demonstrated that *E1* may control the LJ trait (Lu et al 2017), the population in this study was generated, where the segregating population contrasted for *J* and *j−x* and segregated for a single gene controlling the LJ phenotype (Fig. 4). Since a single gene is controlling late flowering and maturity in the previously presumed *e6* lines, and since those lines contain a novel allelic variation at the *J* locus, we conclude that E6 and J are the same locus. The *j-x* allele in Paranagoiana results from a large Ty1-*copia* retrotransposon in the fourth exon of *J* resulting in early termination of translation of the *j−x* protein based on the cDNA results in Supplementary Figure 1. This information will be useful for the development of LJ tropical soybean cultivars.

## Supplementary Information

Below is the link to the electronic supplementary material.

Supplementary file1 (PDF 129 kb)

Supplementary file2 (PDF 112 kb)

Supplementary file3 (xlsx 23 kb)
