# Supplementary material for: Identifying new variation at the J locus, previously identified as e6, in long juvenile ‘Paranagoiana’ soybean
Source: Theor Appl Genet. 2021 Jan 2;134(4):1007–14. doi: 10.1007/s00122-020-03746-2 (PMC7973924; doi:10.1007/s00122-020-03746-2)
Supplement: Supplementary file 1 — Supplementary file1 (PDF 129 kb) [file 122_2020_3746_MOESM1_ESM.pdf]

Identifying New Variation at the J locus, Previously Identified as e6, in 'Paranagoiana' soybean

Nour Nissan[1,2], Elroy R. Cober[1], Michael Sadowski[1,2], Martin Charrette[1], Ashkan Golshani[2], Bahram Samanfar[1,2*]

[1] Agriculture and Agri-Food Canada, Ottawa Research and Development Centre, Ottawa, ON, Canada.
[2]Department of Biology and Ottawa Institute of Systems Biology, Carleton University, Ottawa, ON, Canada.
[*]Corresponding author, bahram.samanfar@canada.ca

Supplementary File 2. Genomic Sequence inserted within exon 4 of *j-x* obtained by primer walking approach. Highlighted in grey are the flanking regions from Glyma.04g050200; underlined regions are the LTR's of the retrotransposon.

TCAACAGAGAGTGTTTGCTGTCCAAGTGTTTGAGTTGCATAGACTGATAAAGGTAAA
TTATCTCTGGCACTGTTCAGCATCTCGTGTTAGTCCAAAATGGGAAGCTTATTAGCTT
TTCTTGCACACACACTCTTGGAGTCTGTTCATACTGATTAATTCCTTTTTATTTAAGC
AGGTCCAACAGCTAATTGCTGGATCACCAGATATTTTGCTTGAAGATGGCGCTTTTC
TGGGAAAGTCTCCTCCAAAGGGATCTATGTAAAAATTCTTGAGGGTTTCCTAGACTA
TCAATTATAGAAAACCAACCGGATCTTGAAATTTATGGTTCTCACAAAACAATCAAT
AAAGAATAATAGATAATTATGTGTACCTTTCTCCATAGGATCTTCTCTCTGGTGCACT
TTGATTTCCTTGAAAATGAGAGAAATAGGATTTCACTTCCTTTGGCCTTTCTTTTATG
TCTCTCTCCGTTCGTGATGGCTATGGGTGAGAAAAGAGCACTTTCTGGTCAGGAAGG
GGACCTTATTTCACATTAGTGGGTATTAAGCCCCTTTTATAACCACTACTCCCATCAA
GTAGCAGTTAATCTAGAAACTTCTCCTATTAAGCCCAATTACAATTTAGCCCTTAATT
GTTAATTATTTATTTATTAGTCCCTACTAAGTCATATGCCTCTCACATGAGACATTAA
TTCTAACATTCTCCCACTTGGCTCATGTGACATTAATAAACATTATGGACTAAATAA
ATAAATAGATTAACTAACATAATGCGCATTAAAAATTGACTAAATTGTTTTACACAT
TGGGTACATCATAATTTCATGATTAAGAATAGGAACCAATTCGAGTCATGGCGGTTG
TACATCATCTAATCGACATAGTCCCTTCCATGTACTACAAATTAGTCTTCTCCTTAAT
ATGACCATTAGTTAGGAGTACATAATTGGTCCAACATAATGTCTTCATTCAAGACAA
AACCTGTTAACATTACTTTAACACAAACATGCATGCAAACATAGAGAACAGGTAAT
CAGAAACATATCATAATTGAGCTCAGAGTGTCAGCAAAATTATATAAGGTAAATTA
CACTTAATGATCATCAATAACAATAATGCCCATACTTTCAACATGTTCTATAAATGT
CTTGGGCGGTAATCCCTTTGTCAAAGGGTCAGCTATCATAAGCTTTGTGTTAATATGT
TCTATTGACACTCTTTGTTTCTGAACTTCTTCTTTCACGGCAAAGTACTTCAATTCCAT
ATGCTTAGCACCCGTAGAGTACTTGTCGTTCTTAGAAAAGAATACTGCTGCGGAGTT
ATCACAATACATTTTCAGTGGCCTAGCAATACTGTCGACAATTCCAAGCCCTGAAAT
AAAGTTCCGCAGCCAATTAGCCTGAATTGTAGCCTCAAAACATGCTACAAATTCAGC
TTCCATGGTGGATGCAGCAACAACTGATTGTTTGCACTCTTCCATGATATTGCTCCT
CCGGCTAAGAGAAATACAAAGCCAAGAGTGGATTTTCTTGTATCCACACATCCAGC
AAAGTCTGAGTCTGAATATCCAATCACCTCTAGGTGATCAGACCTCTTATATGTAAG
CATGTGGTCTTTTGTTCCCTGTAAGTATCTCAGAACTTTCTTTGCAGCTTTCCAATGTT
CCATTCCTAGATTACTTTGATATCTTCCCAACATTCCGGTTGCAAAGCTTATATCTGG
TCGAGTGCAGGTCTGAGCATACATAATACTCCCAACAACTGATGCATACGGAATTGC
TTCCATTTGCTTCCGTTCCAGATCATTTTAGGACATTGTGCGAGACTAAATTTGTCT
CCTTTCTGAATTGGAACGGGAGATGCTGAGCACTTTTCCATCCTAAATCTCTCTAGTA
CTTTATTGATATATGCCTTTTGAGACAAGCCTAACAATCCTTGTGATCTATTTCGGAA
TATTTCTATCCCTATCACATAGCTTGCCTCACCCATATCCTTCATTTCAAAGTTACTA
GAAAGAAACTTCTTAGTCTCATGAAGAAGACCAAGATCATTAGTTGCAAGCAATAT
ATCATCAACATACAGGACTAGAAACATAACCTTACTCCCACTGACCTTCAGATATAC
ACACCGATCAACAGTGTTTTCCTTAAATCCAAAGGAAACAATGGTATCATTAAATTT
CAAATACCATTGGCGGGAAGCTTACTTAAGACCGTATATTGATTTCTTTAATTTGCA
CACCATGTGTTCCTTCCCTTCAACTGAGAATCCCATTGGTTGGTCCATATAAACATCC

TCCTCTAAATCTCCATTCAAAAAGGCAGTTTTCACATCCATCTGATGTAGCTCCAAGT
CATAATGGGCTACTAATGCCATGATAATCCTAAAAGAATCCTTTCGTGAGACTGGTG
AAAACGTCTCTTTATAATCAATGCCATCTTTCTGAGTAAATCCCTTAGCAACAAGTCT
AGCCTTGTAACGTTCAAGGTTGCCATGAGAGTCACGTTTAGTCTTGAAGACCCACTT
ACAACCAACTCTCTTACAACCCTTTGGTAATTCTACAAGGTCCCAAACATCATTATG
TTCCATGGAATCTATCTCTTCTTTCATGGCATTTAACCACTTCTCAGAATTATCACAA
CTTATAGCTTGTGAAAATGAAACTGGATCATTATCATTAATGCTTAAGTTTGTTTCTG
TTTCATGTAAGTATACCACATAGTCATTCGAAATAGCTGGTCTTCTTTCTCTTTGAGA
CTTCCTTAATGCTACTTCCTGTGGTTCTTCCATAATGGGTTCATAATGTATCATGGGC
TCATCATTGTGTTGTACTTCTTCATTACTGTTTGTAGCAGTAACTGAAGTAGTAATCA
CCTTACTGCTAGAGGAAAAAGCTAAAGGGACTTGCACTCTAACTTCTTTAATTTCCA
CTTCTCGTGGAACTGTACTCCCACTGATTTCACCGTTTTCAATGAACCTTGCATTTCC
AGTTTCGACAATTCTCGTACTATGATTAGGACAATAAAACATATACCCCTCTGACTT
TTCTGGATAACCAATGAAATATCCACTGATTGTTCTTGCATCCAATTTTCTTTCTTGC
GGATTGTAAATTCTTATTTCTGCCTGGCAACCCCAAACGTGCAGGTGCCTCATACTA
GGTGTCCTATTTGTCCACAGTTCAAAAGGTGTCTTTGGAACTGCCTTACTAGGAACC
CTATTTAACAAATACATGGCAGTTTTCAAAGCATACATCCACAAAGATACGGGCAA
GGTTGAATAGATTAACATACTCCTAACCATATCCATTAAAGTTCTGTTACGCCTTTCT
GATACACCATTTTGTTGTGGCGTACCAGGCATTGTGTATTGCGCACAAATGCCTCGT
TTCTGAAGGAGCTTAGCAAATGGACCTGGATGTTGCCCAGTTTCATCATATCTTCCG
TAATACTCACCACCTCTATCAGATCTAATAATTTTCACCTTTCTGTCTAATTGTCTTTC
TACTTCATTCAAGTAAATTTCCAAAGCATTCACTGCCTGAGATTTCTCATGCAGTAA
GTAGACATAACCATAACGTGAATAATCATCAATAAAGGTGATAAAGTATCTTTCCCT
TCCGAAAGAACTAACATCAAAAGGTCCACAAATATCTGTATGCACAATTTCAAGAA
GCTGAGTGCTTCTTGTGGCTCCTTTCTTTGTGTGTTTTGTTTGTTTTCCCTTGATACAA
TCCACACAAATATTTAGATCCGTAAAATCTAGATCAGGAAGAATTTCATTCTTTATT
AATCTTTCTATCCTCTCTCTAGAAATGTGACCTAAACGTTTATGCCACAAGAAAGCA
GATCGTTCATTCACTAAACTACGTTTAGTGCCAACATTATGATGCAGAGTTAAAACG
GTTTCAACATACAAACCATCCAATTTCAATTTATATAAACCATCACAAAGAACACCA
GTACCAATGAGATGATTATACTTAAATAAACTGAAACATCCATTACCAAAATTAAAA
GAGTATCCAGTAATATCAAGTTTAGATAATGAAACCAAATTCCTAGATAAACTAGGT
ACATAAAGAGTTTCCAGTAAATCTAAATGATGTACAGTGTCGAGTTTTAAACGATAA
GTCCCGACCGCTTCCACTGGAGCTTTCACTCTATTCCCCATGAAAACAAACTTCTCAT
TTGGGCTTATGGTCTGGATTGTAAGGAATCCCTGCATAGTATTAGAAACATGAGTTG
TACATCCAGAATCAATCCACCATGTATTATGGGGAACTTCAGTTAAGTTTGATTCAA
AACATACAAGAGCATTGAGCTCACCTTTCTTTTCGAACCAAGACTTACGCTTTGGGC
AATCTTTCTGGAAGTGTCCAGATTTTCCACAAAAATGACAATTATTGTTCTTTGATGC
TTTCTTCTGGATTTGCACAGGACCGTCCTTGATCTTTAATGGTCCTTTGCCTTTATCAT
GTTTCTTTACAAATTTTTTTCCAGCTCCTTGATTCCCTCGGTGGCTTACATAATGGATT
GAGTGACTTCCTTGATTCTTAAGCCTCGTTTCTTCCTGAACTAACATACTGTGCAATT
CATGCACATTCCATTTATCTTTCATGGTATTATAGCTCATTTGAAACGGGCCATACTC
AGACGGTAATGAGTTTAGAATAAACTGAACAAGGAAGTTCTCATTCACAGCCATTCC
CAAGGTCTTAAGTCTTGCTGCAATGTTTGTCATCTCAATGACATGTTCATGCATAGTA
CGTGAACCATCAAACTTCATGGTGGTTAGTGTACTCATTAATGTCCCAGCAAGAGAC
TTATCAGCTGTTTGAGAGTGCTCTCCCACTAACCCCATAAACTCTTTAGCACTATCGG
TTTTAGGGAGAGCTGTCTTAATACTGTCTGCAACAGTCATTCTCATGAACATTAGGC

TGAGTCTGTTAGATCTTTCCCAAGCTTTATAATGAGCTTTCTGTTCATTGCTACTAGC
ATCAGTAATAGTAGCAGGCTTCTCTTCCAATATAACAAGGTCAAGATCCAAAACACC
GAGATGAAATTGGACTTGCTCATTCCAATCAGAGAAGTTAAGCCCATTAAAAATTGG
TACAGATGATACATGAGAATTCAATGAGTTGGGAACAGGTACCGCATAATAAAACT
TCACATAAGCATTTTGGTACATGAAACACAAGTCATACATATAATTTACTTAGATAA
AAATCAATGTATATTGATGTTCTCCTTTGGGTGACACACCAACACACAACATACAAA
CATGATGATGCTAATAAAATTTTAACATTATTTGACAATTAAATATGCACCAATTAG
TAGTATCTATTTCCTTTGGGTATATAAATAAAACTAATGATACACACAAATCGCCTA
CAATAATATTCATTAATTATAAGAACAACTAATCAACCTTTGGGCGATCCATAAATG
CCTTATAACAATGAATTTCAATTACCCATAAGCCAACAATCATATAATTTAGCATCC
ATTATTCTATGAGTAATTGAAATAATTATTAATTTGGAATTAAATAACCTTACAAATT
TGGTCACTTTGGTGACTAACAAATTTAACATACATTTAATTCCAACCAAATATATAT
GGCCTGCACATACTTATTGCTATTAACAATTCATAGCCATTCCATTAATTTCATTCTA
GGCCATAAAATAATATTTGCATTTATTTGTGTTTTACATTCTAACACATTCAAGCAAA
TATATAGTATATACATATATTTTACTGCTACCAATAATTGTAAAACATTCACATTAAT
TTAATTATAGAACATAAACTTTCAATCCTTTAATCATGTTTAATGATCATTTTTTGAG
AGAAAAAAAAAATAGGCAAGTGGGATTGTAAATAGCAGAAAGGGGTTTGCAAATA
GAAATTCGAAAAGGAATCCAAACCCAACGTACATAGCAGCACCACTTTTCACAAAA
CTGAAATTAGCACGTACCTACGTGCAGCTTTTGAAAAAGGATAATATTTGCATTTTC
CAAAACGTAATTGGAATTAAAACTTTCTATAAAGTGGTACGTAATTGCCCAATTTTA
TTATTATTATAATATATGATTGTCATCTTTGATCTTAAATAAAAGGCACGAATTTGCA
GTTCGTGATTAAAGTGTCACTTTTGGCTTTCTTTCCCAAAATATAATTTCCAAAAATT
CTATCACTTTCATGGCTTTCCTTCTCATGAAATTTTATATATCTAGAATTTCATACTTT
GCAGCGGAAAATATAATTAACACGAAAGGCATGTTATCAAAACATGTAATTGAAAA
TACATGTAACATAAATTAAAAATCCCTAAATTTCATAATTAGGGTTTATGCATAATT
GGGAGAAATTAAATCATTCTTGGAGAATCATAAATTTCATAACACATGCTCTGATAC
CACATGTAAAAATTCTTGAGGGTTTCCTAGACTATCAATTATAGAAAACCAACCGGA
TCTTGAAATTTATGGTTCTCACAAAACAATCAATAAAGAATAATAGATAATTATGTG
TACCTTTCTCCATAGGATCTTCTCTCTGGTGCACTTTGATTTCCTTGAAAATGAGAGA
AATAGGATTTCACTTCCTTTGGCCTTTCTTTTATGTCTCTCTCCGTTCGTGATGGCTAT
GGGTGAGAAAAGAGCACTTTCTGGTCAGGAAGGGGACCTTATTTCACATTAGTGGG
TATTAAGCCCCTTTTATAACCACTACTCCCATCAAGTAGCAGTTAATCTAGAAACTTC
TCCTATTAAGCCCAATTACAATTTAGCCCTTAATTGTTAATTATTTATTTATTAGTCC
CTACTAAGTCATATGCCTCTCACATGAGACATTAATTCTAACAATCTACTCCCAAAA
AACTTGCACTTGAATATGTTGTAAAACCTCGGCAACAAAACCTTAAGCGCAAAGAT
GATTCTGAAAAGCTAAATCATAAAATGGAATGTTCTGCAGAGAATGCGGTTGGCAA
AACATCTCTTTCATCTGTGAAAGACGGTAGCCACCTTTCAAAATGCACCCCTTTCCCT
GGAAATCAGCACCAGACAAATGTGGCTGCTGACAGTGGGATGGGTCCCTGGTGTTT
CAATCAGTCACCACCTGGGCACCCGTGGCTAATTCCTGTTATGACTCCTTCTGAAGG
ACTTGTCTACAAGCCATATCCGGGGCCTGGATTCACAGGAACTGGTTGTGGAGGAG
GATGCGGGCCTTTTGTGCCTGCTCTGTTGGGTGGTTCTTTCATGAATCCTGGTTATGG
AATCCCAACTTCTCATCAAGGAGTTGGGGTTCCACCAGACACTCATCCTGGCAGTCA
TGGTTACCTCCCTCCATATGGCATGCCAGTTATGAATTCATCAATGTCAGAGTCAGTT
GTTGAACAGGGGAACCAATTCTCTGCACTAGGTTCTCACGGGCATAATGGTCATTTA
CCCGGAGGAGGGAAAGCCAATCATAACACAAACAACAAAAGCTCTTGTAATTTACC
AGTTCAGAGAAATGGAGCAATATCGCATGTCCTGAAACATCAGACATCTAAGGATT

TTGAGTTGCAGGAGACTTCAGCCAGTAGTCCTAGTGAAATGGCACAGGGATTAAGC
ACTGGGCAAGTTGCAGAAGGAAGAGATGTTCTTCCTCTTTTCCCTATGGTTCCAGCA
GAACCAGAGTCTGTTCCTCAGTCTCTTGAAACTGGACAGCATACGCGAGTGATCAAA
GTCGTGCCTCATAACCGAAGATCTGCAACTGCATCAGCAGCTAGAATTTTCCAATCA
ATTCAAGAAGGGAGAAAACAGAATGACTCAGGTAGTGAT
