# Supplementary material for: Identifying new variation at the J locus, previously identified as e6, in long juvenile ‘Paranagoiana’ soybean
Source: Theor Appl Genet. 2021 Jan 2;134(4):1007–14. doi: 10.1007/s00122-020-03746-2 (PMC7973924; doi:10.1007/s00122-020-03746-2)
Supplement: Supplementary file 2 — Supplementary file2 (PDF 112 kb) [file 122_2020_3746_MOESM2_ESM.pdf]

Identifying New Variation at the J locus, Previously Identified as e6, in 'Paranagoiana' soybean

Nour Nissan[1,2], Elroy R. Cober[1], Michael Sadowski[1,2], Martin Charrette[1], Ashkan Golshani[2], Bahram Samanfar[1,2*]

[1] Agriculture and Agri-Food Canada, Ottawa Research and Development Centre, Ottawa, ON, Canada.
[2]Department of Biology and Ottawa Institute of Systems Biology, Carleton University, Ottawa, ON, Canada.
[*]Corresponding author, bahram.samanfar@canada.ca

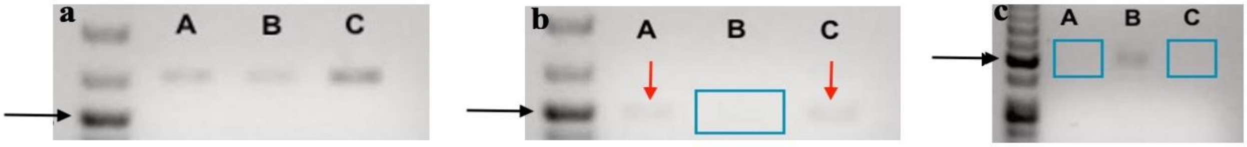

Supplementary Fig. 1 cDNA amplification of Glyma.04g050200 in A) Harosoy (control), B) Paranagoiana (*j-x*) and C) Parana (control) **a** PCR product for the first half of *J* prior to insertion showing ~715 bp bands **b** PCR product for second half of *J* encompassing the insertion in *j-x* line ~492 bp bands (controls lines) and no band for (*j-x* line) **c** PCR product showing ~500 bp of insertion in *j-x*, and no bands present for controls. The blue rectangles highlight absence of bands. Red arrows point to bands. Black arrows point to 500 bp band in ladder. Samples in a and b ran on a 1% agarose gel with GeneRuler 1 kb Plus DNA ladder while samples in c ran on a 2% agarose gel with GeneRuler 50 bp DNA ladder. Gels were imaged using SYBR safe and blue light
